# Seasonal and Spatial Variability of Phytoplankton Primary Production in a Shallow Temperate Coastal Lagoon (Ria Formosa, Portugal)

**DOI:** 10.3390/plants11243511

**Published:** 2022-12-14

**Authors:** Rita B. Domingues

**Affiliations:** CIMA—Centre for Marine and Environmental Research, Campus de Gambelas, University of Algarve, 8005-139 Faro, Portugal; rbdomingues@ualg.pt; Tel.: +351-289-800-900

**Keywords:** phytoplankton production, photosynthetic parameters, light, shallow coastal lagoon, Ria Formosa

## Abstract

Coastal lagoons are among the most productive ecosystems in the world, and they provide a wide range of ecosystem services and resources. In the Ria Formosa (southern Portugal), phytoplankton production has rarely been addressed. The main goal of this study is thus to evaluate the variability of phytoplankton production and photosynthetic characteristics over the seasonal cycle and in different locations (landward, urban, intermediate, and seaward boundaries) of the Ria Formosa coastal lagoon, subjected to distinct natural and anthropogenic stressors. Primary production was evaluated using the ^14^C incorporation technique, and photosynthetic parameters were estimated by fitting photosynthesis-irradiance curves. Primary production showed significant seasonal variations, with higher values in the summer associated with lower euphotic depths, higher water temperatures, and higher nutrient concentrations. No spatial differences were found for primary production or photosynthetic parameters. Primary production values were lower than previous estimates, which reflects an improvement in water quality in the Ria Formosa, but values are higher than primary production estimates for other temperate coastal ecosystems, which reflects the highly productive nature of this coastal lagoon.

## 1. Introduction

Coastal lagoons are shallow brackish or marine water bodies separated from the ocean by a barrier, such as islands, spits, sand banks, or reefs, and connected to the ocean by one or more inlets that remain open at least intermittently [1,2]. Coastal lagoons are usually oriented with a longer dimension parallel to the shore, being much longer than they are wide [3], and with surface areas ranging from a few tens to thousands of square kilometers [4]. Coastal lagoons are a common feature of low-lying coasts, with over 32,000 lagoons reported along 13% of the world’s coastline [3,4].

Coastal lagoons are socio-ecological systems, important from both the ecological and the socio-economic perspectives, providing many valuable ecosystem goods and services, with societal, heritage, aesthetic, and scientific value [5]. Coastal lagoons are also among the most threatened ecosystems, with anthropogenic impacts escalating due to population growth and associated land use alteration [3]. The main pressures to which coastal lagoons are subjected include loss of wetland habitat, changes in connectivity affecting hydrology and sedimentology, contamination, and pollution, all of which threaten the sustainability of these ecosystems [6]. Socio-economic transformation processes, climate change, and eutrophication are ongoing threats that will further impact the structure and function of coastal lagoons, as these ecosystems are highly vulnerable to perturbation [5].

Eutrophication in coastal lagoons is a major problem due to restricted flushing and long water residence times [3]. Eutrophication symptoms include the occurrence of harmful algal blooms, hypoxia and anoxia, increased mortality of benthic organisms, fish mortality, degraded water quality, and an overall alteration of ecosystem structure and function [3]. However, the first consequence of nutrient over-enrichment, which ultimately leads to eutrophication, is the enhancement of primary production. Eutrophication has been defined as “an increase in the rate of supply of organic matter to an ecosystem, which most commonly is related to nutrient enrichment enhancing the primary production in the system” [7]. Therefore, the evaluation of primary production is critical to assessing the eutrophication status of any ecosystem, but particularly in coastal lagoons, which are among the most productive ecosystems in the world [3]. Indeed, due to the high external supply of nutrients from land and from the ocean (in coastal upwelling areas) and its rapid mineralization, and an euphotic zone that extends to the bottom, coastal lagoons support vast communities of primary producers, including seaweeds, seagrasses, microphytobenthos, and phytoplankton [3].

Phytoplankton are usually the main source of organic matter for higher trophic levels in most aquatic ecosystems. In addition to their critical ecological function as primary producers, phytoplankton play pivotal roles in the ecological and biogeochemical dynamics of marine ecosystems influenced by connectivity to land [8]. Phytoplankton are also sensitive indicators of environmental variability, both natural and human-induced, and they have been extensively used as a gauge of ecological condition and change [9]. In coastal lagoons, phytoplankton are undergoing marked changes in biomass, composition, phenology, and production [10], due to anthropogenic and climatic stressors. Phytoplankton production also shows natural spatial and seasonal variability, related to changes in environmental variables such as temperature, light intensity, nutrient availability, also depending on species composition and pigment content [11,12]. The determination of phytoplankton production and its relationship with environmental variables is thus essential to understand ecosystem dynamics and to predict the potential impacts of natural and anthropogenic stressors on ecosystem functioning.

Phytoplankton production can be easily determined using the carbon-14 incorporation method described by Steeman-Nielsen [13], which is currently the standard technique for the determination of primary production [14]. Although a consensus has not yet been reached regarding what is really measured with this technique (gross primary production, net primary production, net community production, or something in-between: [15]), primary production measurements made with ^14^C are closer to net rather than gross primary production [14]. The measurement of carbon incorporation by phytoplankton under different light intensities allows the determination of a light-response curve of photosynthesis, usually known as a photosynthesis-irradiance (P-E) curve, which describes the variability of photosynthetic characteristics of phytoplankton over a range of light conditions. P-E curves are extremely helpful to understand the physiology and ecology of natural phytoplankton communities [16], and they have been the subject of many studies aiming to understand the environmental controls of primary production. The variability of photosynthetic parameters is usually related to variability in environmental drivers, such as water temperature [17], light intensity [18], and nutrients [19], as well as species composition [20] and endogenous circadian rhythms [21]. Therefore, variability in P-E curves is observed at different time scales, especially seasonally [22,23,24], due to the natural variability of light and temperature. These changes in phytoplankton production over seasonal cycles will obviously affect the productivity of the ecosystem and the availability of organic matter to higher trophic levels.

The Ria Formosa coastal lagoon (southern Portugal) is one of the most important aquatic ecosystems in Portugal, both biologically and economically. It serves as a breeding and feeding ground for fish and birds, and supports a wide range of human activities, such as tourism, fishing, and shellfish farming [25]. For instance, 88% of bivalve production in Portugal comes from the Ria Formosa, supporting 7000 families [5]. Due to its importance as a socio-ecological system, water quality and eutrophication in the Ria Formosa have long been addressed, but results have not been consistent. The Ria is subjected to several anthropogenic sources of nutrients, such as discharges of treated domestic and industrial sewage, and runoffs from golf courses and agriculture, which have been associated with increasing nutrient concentrations in the lagoon [5,26]. HABs and non-harmful phytoplankton blooms have been observed in tandem with other eutrophication symptoms, such as water deoxygenation, anoxic sediments, opportunistic green macroalgae blooms, decreased benthos and fish biodiversity, and fish kills; these indicators have been associated with large inputs of N and P, and N:P and N:Si ratios that deviated from the Redfield ratio [5]. However, recent studies suggest that the Ria Formosa is not in a poor condition regarding eutrophication. Neither persistent large inputs of N and P to the Ria nor unbalanced N:P ratios were identified [27], and high semidiurnal tidal prisms promote the flushing of nutrients and contaminants to adjacent waters [28,29,30]. Eutrophication symptoms in the Ria Formosa are only observed in the vicinity of wastewater and industrial discharges, and the low water residence time of the lagoon contributes significantly to its robustness [25].

The previous eutrophication studies in the Ria Formosa coastal lagoon were mostly based on the analysis of nutrient concentrations and ratios, and chlorophyll-a concentration. These indicators are rather static in relation to the dynamic nature of eutrophication, which includes enhancement of primary production in its definition. Measurements of primary production in the Ria Formosa, however, are scarce and somewhat conflicting. Regarding the relative contribution of each primary producer, different studies indicate either salt marsh plants, microphytobenthos, or phytoplankton as the main primary producers (see [25] and references therein). Regarding assessments of phytoplankton primary production, only a few published studies provide phytoplankton production values, using the carbon-14 incorporation method [31] or the oxygen production method [32]. There is indeed a significant lack of knowledge on phytoplankton primary production in this important coastal lagoon, as most published information on phytoplankton in the Ria Formosa addresses only the spatial and seasonal variability of phytoplankton composition [33,34,35] and the effects of nutrient enrichment on phytoplankton community structure [27,28,32]. This study aims, therefore, to evaluate the variability of phytoplankton production and photosynthetic characteristics over the seasonal cycle and in different locations (landward, urban, intermediate, and seaward boundaries) of the Ria Formosa coastal lagoon, subjected to distinct natural and anthropogenic stressors.

## 2. Results

### 2.1. Environmental Conditions

**Figure 1:**
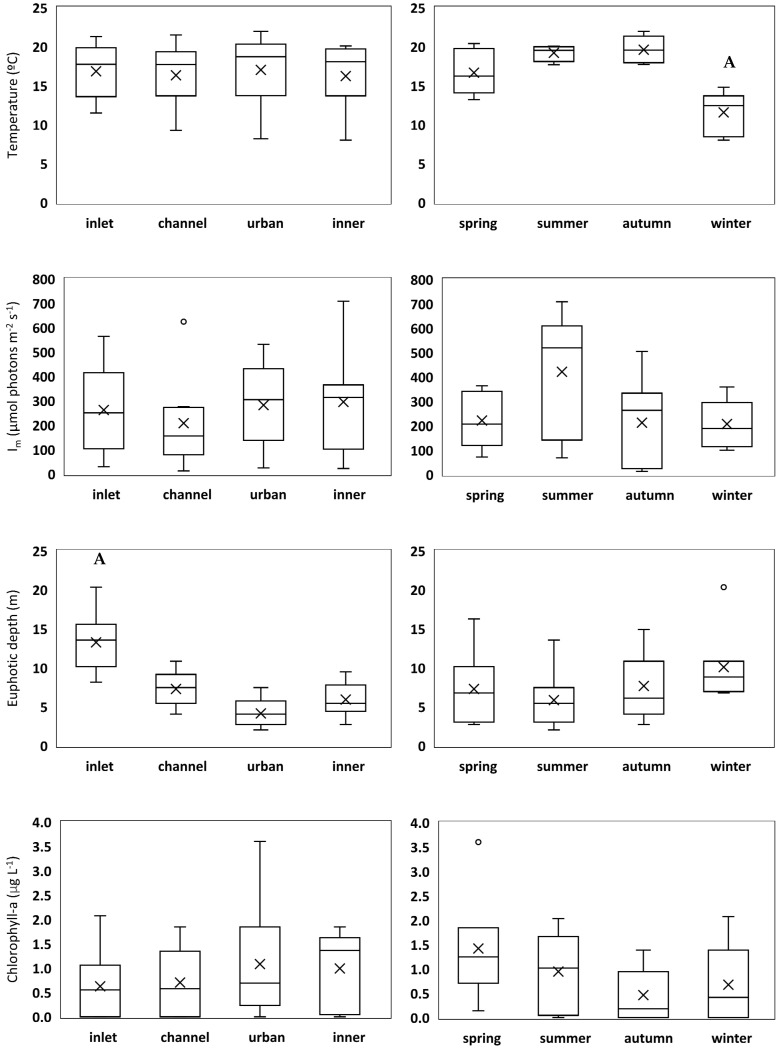

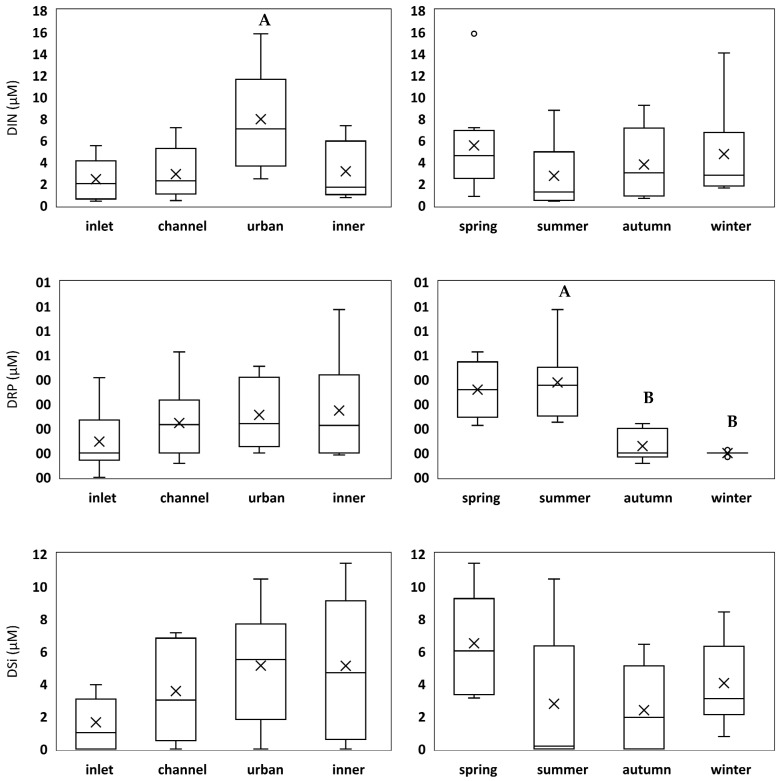
Box−whisker plots showing the median (horizontal line inside the box), mean (cross), interquartile ranges (25th and 75th; boxes), minimum and maximum values (whiskers), and outliers (open circles) for several environmental variables, across sampling locations (left column) and seasons (right column). Significant differences are indicated with letters (A, B) above the respective bar.

Descriptive statistics for different abiotic and biotic variables across seasons and locations are presented in Figure 1 and Tables S1 and S2, and refer to the period May 2011 through April 2012. Considering the whole sampling period and the four sampling locations, water temperature varied between 8.0 and 22.0 °C (mean = 16.6 ± 4.0 °C), and mean light intensity in the mixed layer varied between 12.8 and 708.6 µmol photons m^−2^ s^−1^ (262.1 ± 183.3 µmol photons m^−2^ s^−1^). The depth of the euphotic zone varied between 2.0 and 20.3 m, with a mean value of 7.7 ± 4.4 m. Mean dissolved inorganic nitrogen (DIN), dissolved reactive phosphorus (DRP), and dissolved silicon (DSi) concentrations throughout the sampling period were 4.2 ± 3.8 µM, 0.2 ± 0.2 µM, and 3.8 ± 3.4 µM, whereas chlorophyll-a concentration ranged between undetectable values and 3.6 µg L^−1^, with a mean value of 0.85 ± 0.85 µg L^−1^. Spatial differences were found for euphotic depth (*p* < 0.001), with significantly higher values at the inlet (seaward boundary), and for DIN (*p* = 0.002), with significantly higher concentrations at the urban location in relation to the other three locations (Figure 1, Supplementary Table S2). Seasonal differences were found for water temperature, with winter values significantly lower than values registered during the other seasons (*p* < 0.001), and for DRP (*p* < 0.001), with significantly higher concentrations in the summer and lower in the autumn and winter (Figure 1, Supplementary Table S1).

### 2.2. Primary Production and Photosynthetic Parameters

**Figure 2:**
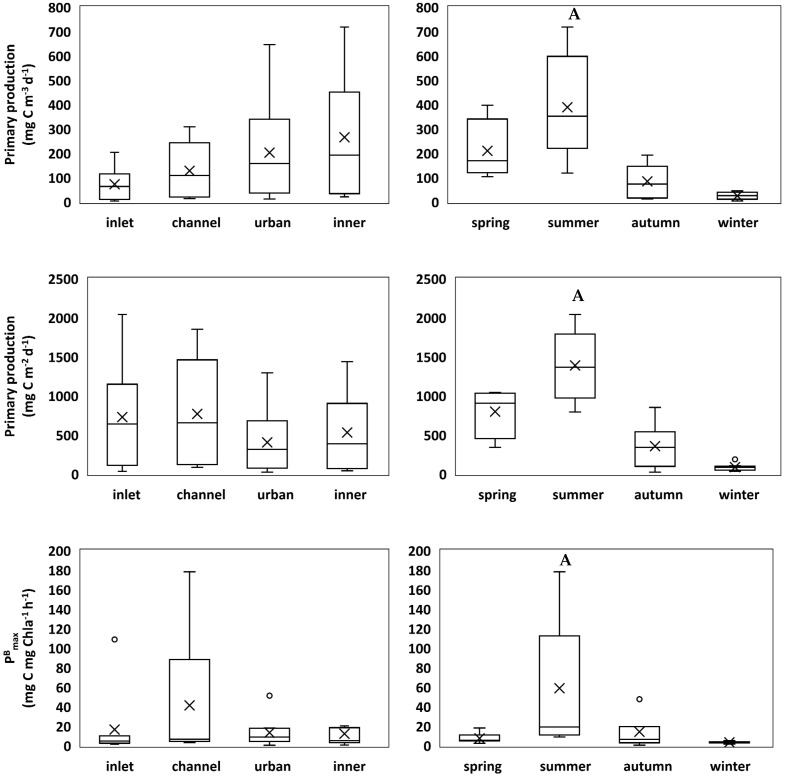

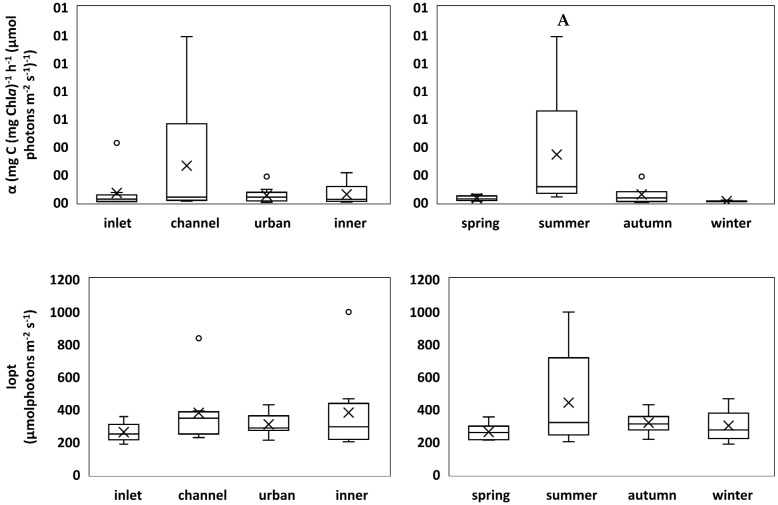
Box−whisker plots showing the median (horizontal line inside the box), mean (cross), interquartile ranges (25th and 75th; boxes), minimum and maximum values (whiskers), and outliers (open circles) for volumetric primary production, aerial primary production, maximal biomass-specific primary production rate (P^B^_max_), photosynthetic efficiency (α), and optimal light intensity (I_opt_), across sampling locations (left column) and seasons (right column). Significant differences are indicated with letters (A) above the respective bar.

Primary production in the euphotic zone between May 2011 and April 2012 varied between 3.51 and 718.74 mg C m^−3^ d^−1^, with a mean value of 167.1 ± 188.7 mg C m^−3^ d^−1^. Areal primary production ranged between 23 and 2042 mg C m^−2^ d^−1^, with a mean value of 610 mg C m^−3^ d^−1^ (Figure 2). Significant seasonal differences in primary production were found, with summer values significantly higher (*p* < 0.001) (Supplementary Table S1). The same trend was found for the photosynthetic parameters maximal biomass-specific production rate (P^B^_max_) and photosynthetic efficiency (α). P^B^_max_ varied between 0.5 and 178.3 mg C mg Chl*a*^−1^ h^−1^ (20.6 ± 38.6 mg C mg Chl*a*^−1^ h^−1^) and α presented values ranging between 0.00 and 1.19 mg C mg Chl*a*^−1^ h^−1^ (µmol photons m^−2^ s^−1^)^−1^ (0.11 ± 0.24 mg C mg Chl*a*^−1^ h^−1^ (µmol photons m^−2^ s^−1^)^−1^). Both P^B^_max_ (*p* = 0.008) and α (*p* = 0.009) were significantly higher in the summer in relation to the other seasons, but no spatial differences were observed for these parameters. The optimal light intensity (E_opt_) ranged between 186.5 and 998.5 µmol photons m^−2^ s^−1^ (328.6 ± 165.3 µmol photons m^−2^ s^−1^), and no significant spatial or seasonal differences were detected (Figure 2). 

### 2.3. Relationships between Primary Production, Photosynthetic Parameters, and Environmental Variables

Significant moderate to strong correlations were found between primary production and environmental variables. Primary production was strongly and positively correlated with dissolved reactive phosphorus (r = 0.863, *p* < 0.001) and negatively correlated with the euphotic depth (r = −0.463, *p* = 0.010). Significant moderate and positive correlations were also found between primary production and water temperature (r = 0.436, *p* = 0.026) and chlorophyll-a concentration (r = 0.401, *p* = 0.028). Chl*a* was, in turn, positively correlated with DIN (r = 0.425, *p* = 0.012). As for photosynthetic parameters, no significant correlations were found between those and environmental variables, but a strong and positive correlation was found between α and P^B^_max_ (r = 0.977, *p* < 0.001). E_opt_ was also correlated with I_m_ (r = 0.411, *p* = 0.016), α (r = 0.491, *p* = 0.003), and P^B^_max_ (r = 0.469, *p* = 0.005).

The model obtained through redundancy analysis explained 38% of the constrained variance, with an adjusted R^2^ of 0.23 (*p* = 0.028). Euphotic depth (score = −0.70) and phosphorus concentration (score = 0.83) were more strongly associated with the first axis, whereas nitrogen concentration (score = 0.53) and chlorophyll-a (score = 0.78) were associated with the second axis.

## 3. Discussion

In the Ria Formosa coastal lagoon, primary production showed significant seasonal variation, with higher values in the summer, associated with lower euphotic depths, higher water temperatures, and higher nutrient concentrations. No spatial differences were found for primary production or photosynthetic parameters. 

The estimates of phytoplankton primary production in the Ria Formosa (167.1 ± 188.7 mg C m^−3^ d^−1^) are lower than values previously reported for this coastal lagoon. For instance, studies conducted in the early 1990s and the early 2000s reported mean (±SD) primary production values of 389 ± 273 mg C m^−3^ d^−1^ [36], 418 ± 424 mg C m^−3^ d^−1^ [37], and 740 ± 1507 mg C m^−3^ d^−1^ [38]. The strong dispersion of values reflects the seasonal variability in primary production in this ecosystem. The lower primary production values measured in the present study may be associated with a generalized improvement in water quality that has been detected in the Ria Formosa in relation to the early 2000s, due to the upgrade of wastewater treatment systems [30,39]. In addition, high semidiurnal tidal prisms and low water residence time contribute to the lagoon’s robustness and low susceptibility to eutrophication [25,35]. Only one study reported planktonic primary production values much lower than ours, which were probably significantly underestimated; in that study, gross primary production attributed to plankton was 0.00144 mmol C m^−2^ h^−1^ [40], whereas our values are three orders of magnitude higher (mean = 2.05 mmol C m^−2^ h^−1^). 

Overall, the primary production values estimated for the Ria Formosa coastal lagoon (areal primary production ranged between 23 and 2042 mg C m^−2^ d^−1^) are higher than values reported for other temperate coastal ecosystems in Portugal. For instance, areal primary production in Portuguese estuaries ranges between 2 and 778 mg C m^−2^ d^−1^ in the Tagus estuary [24], 10–1140 mg C m^−2^ d^−1^ in the Guadiana estuary [23], and 5–1880 mg C m^−2^ d^−1^ in the Douro estuary [41], the latter closer to values measured in the Ria Formosa. Nevertheless, annual primary production values in the Ria Formosa coastal lagoon (median = 141 mg C m^−2^ yr^−1^; mean = 223 mg C m^−2^ yr^−1^) are similar to values found in a compilation of 1148 values measured in 131 coastal ecosystems, with a median and mean annual primary production of 185 and 252 g C m^−2^ yr^−1^ [8]. The comparison of primary production values must be performed with caution, as different techniques to measure primary production, incubation protocols, and methods for integrating primary production rates over depth and time can yield up to a 3-fold variability in estimates [8]. 

Significant spatial differences in primary production in the Ria Formosa coastal lagoon, with lower values at the inlet, were found in a previous study [38], whereas other studies found no such spatial variability [32]. Our primary production values were also lower at the inlet (53.9 ± 46.9 mg C m^−3^ d^−1^), but the differences in relation to the other locations were not significant (highest values at the inner lagoon: 235.1 ± 277.2 mg C m^−3^ d^−1^). The inlet is the deepest location of the lagoon, with a deeper euphotic zone and lower mean light intensity in the mixed layer. Although the redundancy analysis resulted in a model that explained only 38% of the variability in primary production and photosynthetic parameters, euphotic depth was negatively and strongly associated with the response variables. Deeper lagoon locations, despite a well-mixed water column and a euphotic zone that extends to the bottom, will have a lower mean light intensity. Given that phytoplankton dynamics are strongly associated with light availability, it is expected that primary production will be lower at deeper lagoon locations, such as the inlets. Light is particularly important for phytoplankton in confined and/or turbid ecosystems [42,43], regulating the structure of phytoplankton communities; light limitation commonly occurs in coastal ecosystems, and, thus, primary production rarely reaches its potential [44]. In the Ria Formosa, phytoplankton growth is indeed limited by light at the deeper lagoon locations [28], particularly in the winter [27]. In other temperate coastal ecosystems, photosynthetic activity is also strongly correlated with light availability (e.g., [21]), particularly in turbid, nutrient-rich estuaries (e.g., [23,45]).

The nutritional environment is another major variable regulating phytoplankton growth and production. According to the constrained ordination, both nitrogen and phosphorus were related to the patterns observed in primary production and photosynthetic parameters, with higher nutrient concentrations associated with higher primary production. Co-limitation by N and P is frequently observed throughout the seasonal cycle in the Ria Formosa coastal lagoon [46], and higher nutrient concentrations are associated with higher phytoplankton growth [28]. Nutrient, particularly nitrogen, availability is commonly a major factor regulating primary production and photosynthetic activity in different types of ecosystems [23,47], but is particularly relevant in shallow ecosystems such as the Ria Formosa, where light limitation seldom occurs [27]. 

Regarding photosynthetic parameters, no significant spatial variability was found, and no correlations with environmental variables. The maximal biomass-specific production rate (P^B^_max_) and photosynthetic efficiency (α) were strongly correlated with each other, which is not expected, as α is related to the concentration of photosynthetically active pigments [48] and P^B^_max_ is related to electron transport capacities [49]. However, many studies have observed linear relationships between the two parameters (e.g., [21,24,50]), attributed to a variety of physiological and ecological factors, and changes in phytoplankton composition [51]. The positive relationship between P^B^_max_ and α indicates that phytoplankton with a lower photosynthetic efficiency tends to exhibit a lower maximum photosynthetic rate, which contradicts the typical photo-acclimation of phytoplankton, with lower photosynthetic efficiency but the higher maximum photosynthetic rate at surface waters, associated with higher light availability [52]. The optimal light intensity (E_opt_) was positively correlated with light availability in the water column, as expected, given that the cells will typically adjust their light saturation coefficient in accordance with ambient irradiance [53]. Despite no significant spatial variability or correlation with environmental variables, photosynthetic parameters varied seasonally, with significantly higher P^B^_max_ and α observed during summer. Higher summer values for photosynthetic parameters are typically observed in coastal ecosystems, associated with higher water temperature and light availability [21] and favorable upwelling conditions [54]. In the Ria Formosa coastal lagoon, water temperature in the spring, summer, and autumn periods was significantly higher than in the winter, and upwelling events in the adjacent coastal area are a common occurrence throughout the late spring-early summer period [55]. The coastal upwelling events may enter the Ria and extend approx. 6 km upstream from the lagoon inlets [29], providing nutrients that will stimulate phytoplankton production.

In conclusion, planktonic primary production in the Ria Formosa coastal lagoon showed marked seasonal variability, with the summer months being more productive, associated with higher light intensity and higher temperature but no significant spatial differences were found. Estimates of primary production are within values reported for other coastal ecosystems, but are lower than previous estimates for the Ria Formosa, possibly due to an improvement in water quality in the last decade. Due to the shallowness of the Ria Formosa, a strong benthic-pelagic coupling is expected, and the relative contribution of benthic primary producers to the total productivity of the system should be as relevant as the planktonic component. Future studies should address not only the total productivity of the system but also the relative contribution of each component to overall primary production. Given that the primary productivity of an ecosystem constrains the availability of fish and other commercially relevant organisms, the assessment of primary production is of strategic economic importance in coastal ecosystems [56].

## 4. Material and Methods

### 4.1. Study Site and Sampling Strategy

**Figure 3:**
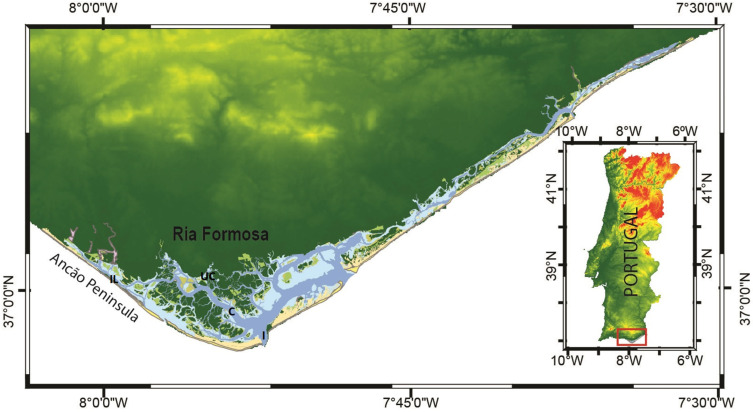
Map of the Ria Formosa coastal lagoon and sampling stations. IL—inner lagoon (landward boundary); UC—urban center (urban boundary); C—main navigational channel (intermediate lagoon domain); I—inlet (seaward boundary).

The Ria Formosa coastal lagoon (Figure 3) is an euryhaline, shallow ecosystem protected by a multi-inlet barrier island system. It is located in southern Portugal and extends c. 55 km E-W and c. 6 km N-S at its widest point. The Ria Formosa is subjected to Mediterranean climate with hot, dry summers, and moderate winters. Tides in the Ria Formosa are semidiurnal and mesotidal, with a mean amplitude of 2.1 m [57]. 

This study was part of research project PHYTORIA, conducted in the Ria Formosa coastal lagoon during 2011 and 2012, focused on spatial and seasonal dynamics of planktonic microbes. For the present study, sampling campaigns were conducted in the following four representative locations in the Ria Formosa coastal lagoon, from May 2011 to April 2012: a more confined location, the “inner lagoon”; a location close to a major “urban centre”; a location in the main navigational “channel”; in the main lagoon “inlet” (Figure 3). These sampling stations were representative of the landward, urban, intermediate, and seaward boundaries, respectively. Mean depth of inner lagoon and urban center stations is 2 m and mean depth of channel and inlet stations is 6 m. The water column is well-mixed, due to its shallowness; occasional thermal stratification can be observed in locations closer to the seaward boundary [36]. Monthly sampling was conducted during mid-ebb tidal stage of neap tides, starting at station “inner lagoon” and finishing at the lagoon “inlet”. Vertical profiles of water temperature and salinity were measured with a YSI 556 MPS probe and vertical profiles of photosynthetically active radiation (PAR) intensity were obtained with a spherical LI-COR sensor. Sub-superficial water samples were collected at each sampling station using a Niskin bottle into 10 L polycarbonate Nalgene bottles, which were transported to the lab in cold and dark conditions (approx. 30 min), and subsequently analyzed for dissolved inorganic nutrients, chlorophyll *a* concentration, and primary production (see Section 4.3 and Section 4.4). All material used in the field and in the lab was thoroughly washed with HCl 10% and rinsed with deionized water. 

### 4.2. Light Measurements

Light attenuation coefficients (k_d_, m^−1^) for each sampling date were estimated using the vertical profiles of PAR intensity, according to an exponential function (Equation (1)) as follows:

(1) $$I_{Z}=I_{0}e^{\left(-k_{d}Z\right)}$$

where I_z_ (µmol photons m^−2^ s^−1^) is the PAR intensity at depth level z (m) and I_0_ is the PAR intensity at the surface. Euphotic zone depth (Z_eu_, m) was calculated as (Equation (2)) as follows:

(2) $$Z_{\mathrm{eu}}=4.61/k_{d}$$

assuming that irradiance at the bottom was 1% of surface irradiance [58]. Mean PAR intensity in the water column (I_m_, µmol photons m^−2^ s^−1^) for each sampling date was calculated as (Equation (3)) as follows:

(3) $$I_{m}=I_{0}\left(1-e^{\left(-k_{d}Z_{m}\right)}\right){\left(k_{d}Z_{m}\right)}^{-1}$$

where z_m_ (m) is the depth of the mixed layer. Given that our vertical profiles of PAR intensity were obtained at a specific time of the day that could not be representative of the whole day [22]. I_0_ was determined using daily solar irradiance data (W m^−2^, converted to µmol photons m^−2^ s^−1^ using a conversion factor of 4.587 mmol photons s^−1^ W^−1^: [59] obtained at https://snirh.apambiente.pt/ (accessed on 15 September 2022), considering that PAR constitutes 50% of the total solar radiation reaching the surface and a mean reflection of 6.6% at the surface [60]. 

### 4.3. Phytoplankton Primary Production

Phytoplankton primary production was determined using the [13]. Fifty mL aliquots of each sample were collected into triplicate polycarbonate flasks and 100 µL (7.4 × 10^4^ Bq) of ^14^C-HCO_3_^−^ were added to each flask. The flasks were incubated in a plant growth chamber under in situ temperature and different light intensities (0, 47, 113, 230, and 365 µmol photons m^−2^ s^−1^), and carbon uptake was stopped with formaldehyde after 2 h. Primary production for each light level was calculated as (Equation (4)) as follows:

(4) $$\mathrm{PP}=\frac{\left(\mathrm{Rs}-\mathrm{Rd}\right)\times D\times W}{R\times N}$$

where PP is phytoplankton primary production (mg C L^−1^ h^−1^), Rs (dpm) is the activity in the sample, Rd (dpm) is the mean activity of the dark flasks, D (=1.05) is the isotopic discrimination (correction for the preference for ^12^C over ^14^C [61]), W (mg C L^−1^) is the amount of dissolved inorganic carbon in the sample (obtained through alkalinity), R (dpm) is the total activity of the ^14^C added to each flask, and N (hours) is the incubation time. 

Primary production (PP, mg C m^−3^ h^−1^) was converted to biomass-specific primary production, divided by the sample’s chlorophyll *a* concentration (P^B^ = PP/Chl*a*, mg C mg Chl*a*^−1^ h^−1^). Photosynthetic parameters were estimated using [62] model through nonlinear fitting of Equation (5) as follows:

(5) $$P^{B}=\frac{E}{aE^{2}+bE+c}$$

where P^B^ is biomass-specific primary production (mg C mg Chl*a*^−1^ h^−1^) and E is the PAR irradiance during incubation (µmol photons m^−2^ s^−1^). Coefficients a, b, and c were used to estimate maximal biomass-specific production rate (P^B^_max_, mg C mg Chl*a*^−1^ h^−1^), optimal light intensity (E_opt_, µmol photons m^−2^ s^−1^), and initial slope of the P-E curve, which represents photosynthetic efficiency (α, mg C mg Chl*a*^−1^ h^−1^ (µmol photons m^−2^ s^−1^)^−1^), according to Equations (6)–(8), respectively, as follows:

(6) $$P_{\max}^{B}=\frac{1}{b+2\sqrt{\mathrm{ac}}}$$

(7) $$E_{\mathrm{opt}}=\sqrt{c/a}$$

(8) α=1/c

Daily volumetric primary production (mg C m^−3^ d^−1^) was estimated by integration over depth in the euphotic zone, using non-normalized P_max_ estimates. 

### 4.4. Other Analytical Procedures

Alkalinity was determined by acidification with HCl 0.01 N [63]. Carbonate alkalinity was then converted to dissolved inorganic carbon and subsequently used in primary production determinations.

Water samples for determination of dissolved inorganic nutrient concentration were immediately filtered after collection through cellulose acetate filters (Whatman, nominal pore diameter = 0.2 μm) to acid-cleaned vials. Ammonium (NH_4_^+^), phosphate (PO_4_^3−^), and silicate (SiO_4_^4−^) were determined immediately upon arrival to the laboratory, whilst samples for nitrate (NO_3_^−^) were frozen (−20 °C) until analysis. All nutrient analyses were made in triplicate, according to the spectrophotometric methods described by [64], using a spectrophotometer Hitachi U-2000 for ammonium, phosphate, and silicate, and an autoanalyzer Skalar for nitrate and nitrite. Chlorophyll *a* concentration was also determined spectrophotometrically, using glass fiber filters and acetone 90% to extract the pigment [63].

### 4.5. Data Analyses

Descriptive statistics were used to explore the data in terms of averages and dispersion. Differences across locations (combining data from all seasons) and seasons (combining data from all locations) were tested with analysis of variance (ANOVA) or Kruskall-Wallis ANOVA on ranks, depending on data normality, assessed with a Kolmogorov-Smirnov test. The strength of associations between variables was measured with Pearson’s correlation coefficient. These analyses were performed using IBM SPSS Statistics v. 28. A photosynthesis-irradiance model [62] was fitted to the light intensity versus non-normalized primary production, PP (for water column production estimates) or normalized primary production, P^B^ (for photosynthetic parameters) data using the function nls on R. Redundancy analysis (RDA) was used to examine relationships between environmental variables and primary production-related variables, to determine which predictor variables explain the most variation in multiple response variables [65]. Analyses were carried out with the “vegan” package [66] for R [67] using the RStudio environment [68]. All analyses were considered at a 0.05 significance level.

## Data Availability

The data presented in this study are available on request from the corresponding author.

## Supplementary Materials

The following supporting information can be downloaded at: https://www.mdpi.com/article/10.3390/plants11243511/s1, Table S1: descriptive statistics across locations; Table S2: descriptive statistics across seasons.

## References

[1] Kjerfve B. (1994). Coastal Lagoon Processes.

[2] Adlam K. (2014). Coastal Lagoons: Geologic Evolution in Two Phases. Mar. Geol..

[3] Kennish M., Paerl H. (2010). Coastal Lagoons: Critical Habitats of Environmental Change.

[4] Carrasco A.R., Ferreira Ó., Roelvink D. (2016). Coastal Lagoons and Rising Sea Level: A Review. Earth-Sci. Rev..

[5] Newton A., Icely J., Cristina S., Brito A., Cardoso A.C., Colijn F., Riva S.D., Gertz F., Hansen J.W., Holmer M. (2014). An Overview of Ecological Status, Vulnerability and Future Perspectives of European Large Shallow, Semi-Enclosed Coastal Systems, Lagoons and Transitional Waters. Estuar. Coast. Shelf Sci..

[6] Newton A., Icely J., Cristina S., Perillo G.M.E., Turner R.E., Ashan D., Cragg S., Luo Y., Tu C., Li Y. (2020). Anthropogenic, Direct Pressures on Coastal Wetlands. Front. Ecol. Evol..

[7] EEA (2001). Eutrophication in Europe’s Coastal Waters.

[8] Cloern J.E., Foster S.Q., Kleckner A.E. (2013). Review: Phytoplankton Primary Production in the World’s Estuarine-Coastal Ecosystems. Biogeosci. Discuss..

[9] Domingues R.B., Barbosa A., Galvão H. (2008). Constraints on the Use of Phytoplankton as a Biological Quality Element within the Water Framework Directive in Portuguese Waters. Mar. Pollut. Bull..

[10] Smetacek V., Cloern J.E. (2008). On Phytoplankton Trends. Science.

[11] Finenko Z.Z., Churilova T.Y., Sosik H.M., Basturk O. (2002). Variability of Photosynthetic Parameters of the Surface Phytoplankton in the Black Sea. Oceanology.

[12] Falkowski P.G., Raven J.A. (1997). Aquatic Photosynthesis.

[13] Steeman-Nielsen E. (1952). The Use of Radio-Active Carbon (C14) for Measuring Organic Production in the Sea. J. Counseil Int. Pour l’Explor..

[14] Regaudie-de-Gioux A., Lasternas S., Agustí S., Duarte C.M. (2014). Comparing Marine Primary Production Estimates through Different Methods and Development of Conversion Equations. Front. Mar. Sci..

[15] Gazeau F., Middelburg J.J., Loijens M., Vanderborght J.-P., Pizay M.-D., Gattuso J.-P. (2007). Planktonic Primary Production in Estuaries: Comparison of 14C, O2 and 18O Methods. Aquat. Microb. Ecol..

[16] Morán X., Estrada M. (2001). Short-Term Variability of Photosynthetic Parameters and Particulate and Dissolved Primary Production in the Alboran Sea (SW Mediterranean). Mar. Ecol. Prog. Ser..

[17] Yoshie N., Suzuki K., Kuwata A., Nishioka J., Saito H. (2010). Temporal and Spatial Variations in Photosynthetic Physiology of Diatoms during the Spring Bloom in the Western Subarctic Pacific. Mar. Ecol. Prog. Ser..

[18] Palmer M.A., Arrigo K.R., Mundy C.J., Ehn J.K., Gosselin M., Barber D.G., Martin J., Alou E., Roy S., Tremblay J.-É. (2011). Spatial and Temporal Variation of Photosynthetic Parameters in Natural Phytoplankton Assemblages in the Beaufort Sea, Canadian Arctic. Polar Biol..

[19] Napoléon C., Raimbault V., Fiant L., Riou P., Lefebvre S., Lampert L., Claquin P. (2012). Spatiotemporal Dynamics of Physicochemical and Photosynthetic Parameters in the Central English Channel. J. Sea Res..

[20] Jouenne F., Lefebvre S., Véron B., Lagadeuc Y. (2005). Biological and Physicochemical Factors Controlling Short-Term Variability in Phytoplankton Primary Production and Photosynthetic Parameters in a Macrotidal Ecosystem (Eastern English Channel). Estuar. Coast. Shelf Sci..

[21] Azevedo I., Duarte P., Bordalo A. (2010). Temporal and Spatial Variability of Phytoplankton Photosynthetic Characteristics in a Southern European Estuary (Douro, Portugal). Mar. Ecol. Prog. Ser..

[22] Domingues R.B., Anselmo T.P., Barbosa A.B., Sommer U., Galvão H.M. (2011). Light as a Driver of Phytoplankton Growth and Production in the Freshwater Tidal Zone of a Turbid Estuary. Estuar. Coast. Shelf Sci..

[23] Domingues R.B., Barbosa A.B., Sommer U., Galvão H.M. (2012). Phytoplankton Composition, Growth and Production in the Guadiana Estuary (SW Iberia): Unraveling Changes Induced after Dam Construction. Sci. Total Environ..

[24] Gameiro C., Zwolinski J., Brotas V. (2011). Light Control on Phytoplankton Production in a Shallow and Turbid Estuarine System. Hydrobiologia.

[25] Barbosa A.B., Kennish M.J., Paerl H.W. (2010). Seasonal and Interanual Variability of Planktonic Microbes in a Mesotidal Coastal Lagoon (Ria Formosa, SE Portugal). Impact of Climatic Changes and Local Human Influences. Coastal Lagoons: Critical Habitats of Environmental Change.

[26] Cravo A., Fernandes D., Damião T., Pereira C., Reis M.P. (2015). Determining the Footprint of Sewage Discharges in a Coastal Lagoon in South-Western Europe. Mar. Pollut. Bull..

[27] Domingues R.B., Guerra C.C., Barbosa A.B., Galvão H.M. (2017). Will Nutrient and Light Limitation Prevent Eutrophication in an Anthropogenically-Impacted Coastal Lagoon?. Cont. Shelf Res..

[28] Domingues R.B., Guerra C.C., Barbosa A.B., Galvão H.M. (2015). Are Nutrients and Light Limiting Summer Phytoplankton in a Temperate Coastal Lagoon?. Aquat. Ecol..

[29] Cravo A., Cardeira S., Pereira C., Rosa M., Alcântara P., Madureira M., Rita F., Luis J., Jacob J. (2014). Exchanges of Nutrients and Chlorophyll a through Two Inlets of Ria Formosa, South of Portugal, during Coastal Upwelling Events. J. Sea Res..

[30] Cravo A., Ferreira C., Jacob J., Saldanha Matos J., Rosa M.J. (2018). Water Quality Improvement in Ria Formosa since the Early 2000. Sanitation Approaches and Solutions and The Sustainable Development Goals.

[31] Domingues R.B., Guerra C.C., Barbosa A.B., Brotas V., Galvão H.M. (2014). Effects of Ultraviolet Radiation and CO_2_ Increase on Winter Phytoplankton Assemblages in a Temperate Coastal Lagoon. J. Plankton Res..

[32] Loureiro S., Newton A., Icely J. (2005). Effects of Nutrient Enrichments on Primary Production in the Ria Formosa Coastal Lagoon (Southern Portugal). Hydrobiologia.

[33] Loureiro S., Newton A., Icely J. (2006). Boundary Conditions for the European Water Framework Directive in the Ria Formosa Lagoon, Portugal (Physico-Chemical and Phytoplankton Quality Elements). Estuar. Coast. Shelf Sci..

[34] Pereira M.G., Icely J., Mudge S., Newton A., Rodrigues R. (2007). Temporal and Spatial Variation of Phytoplankton Pigments in the Western Part of Ria Formosa Lagoon, Southern Portugal. Environ. Forensics.

[35] Cravo A., Barbosa A.B., Correia C., Matos A., Caetano S., Lima M.J., Jacob J. (2022). Unravelling the Effects of Treated Wastewater Discharges on the Water Quality in a Coastal Lagoon System (Ria Formosa, South Portugal): Relevance of Hydrodynamic Conditions. Mar. Pollut. Bull..

[36] Barbosa A.B. (2006). Estrutura e Dinâmica Da Teia Alimentar Microbiana Na Ria Formosa. Ph.D. Thesis.

[37] Galvão H.M., Mendes P.J., Caetano S.M., Icely J.D., Newton A. (2019). Role of Microbes in the Ria Formosa Lagoon. Ria Formosa—Challenges of a Coastal Lagoon in a Changing Environment.

[38] Newton A., Icely J.D. (2006). Oceanographic Applications to Eutrophication in Tidal, Coastal Lagoons: The Ria Formosa, Portugal. J. Coast. Res..

[39] Rosa A., Cravo A., Jacob J., Correia C. (2022). Water Quality of a Southwest Iberian Coastal Lagoon: Spatial and Temporal Variability. Cont. Shelf Res..

[40] Santos R., Silva J., Alexandre A., Navarro N., Barrón C., Duarte C.M. (2004). Ecosystem Metabolism and Carbon Fluxes of a Tidally- Dominated Coastal Lagoon. Estuaries.

[41] Azevedo I.C., Duarte P.M., Bordalo A.A. (2006). Pelagic Metabolism of the Douro Estuary (Portugal)—Factors Controlling Primary Production. Estuar. Coast. Shelf Sci..

[42] Kocum E., Underwood G.J.C., Nedwell D.B. (2002). Simultaneous Measurement of Phytoplanktonic Primary Production, Nutrient and Light Availability along a Turbid, Eutrophic UK East Coast Estuary (the Colne Estuary). Mar. Ecol. Prog. Ser..

[43] Cloern J.E. (1999). The Relative Importance of Light and Nutrient Limitation of Phytoplankton Growth: A Simple Index of Coastal Ecosystem Sensitivity to Nutrient Enrichment. Aquat. Ecol..

[44] Kromkamp J., Peene J., Rijswijk P., Sandee A., Goosen N. (1995). Nutrients, Light and Primary Production by Phytoplankton and Microphytobenthos in the Eutrophic, Turbid Westerschelde Estuary (The Netherlands). Hydrobiologia.

[45] Loken L.C., Sadro S., Lenoch L.E.K., Stumpner P.R., Dahlgren R.A., Burau J.R., Van Nieuwenhuyse E.E. (2022). Whole-Ecosystem Experiment Illustrates Short Timescale Hydrodynamic, Light, and Nutrient Control of Primary Production in a Terminal Slough. Estuaries Coasts.

[46] Domingues R.B., Nogueira P., Barbosa A.B. Co-Limitation of Phytoplankton by N and P in a Shallow Coastal Lagoon (Ria Formosa, Southern Portugal): Implications for Eutrophication Evaluation. Estuar. Coast. Shelf Sci..

[47] Ko E., Gorbunov M.Y., Jung J., Lee Y., Cho K.-H., Yang E.J., Park J. (2022). Phytoplankton Photophysiology Varies Depending on Nitrogen and Light Availability at the Subsurface Chlorophyll Maximum in the Northern Chukchi Sea. Front. Mar. Sci..

[48] Steeman-Nielsen E., Jorgensen E.G. (1968). The Adaptation of Plankton Algae. I. General Part. Physiol. Plant..

[49] Behrenfeld M.J., Halsey K.H., Milligan A.J. (2008). Evolved Physiological Responses of Phytoplankton to Their Integrated Growth Environment. Philos. Trans. R. Soc. B Biol. Sci. R. Soc..

[50] Lamont T., Barlow R.G., Kyewalyanga M.S. (2014). Physical Drivers of Phytoplankton Production in the Southern Benguela Upwelling System. Deep Sea Res. Part I Oceanogr. Res. Pap..

[51] Bouman H.A., Platt T., Doblin M., Figueiras F.G., Gudmundsson K., Gudfinnsson H.G., Huang B., Hickman A., Hiscock M., Jackson T. (2018). Photosynthesis-Irradiance Parameters of Marine Phytoplankton: Synthesis of a Global Data Set. Earth Syst. Sci. Data.

[52] Cullen J.J., Yang X., Macintyre H.L., Falkowski P.G., Woodhead A.D. (1992). Nutrient Limitation of Marine Photosynthesis. Primary Productivity and Biogeochemical Cycles in the Sea.

[53] Sakshaug E., Bricaud A., Dandonneau Y., Falkowski P.G., Kiefer D.A., Legendre L., Morel A., Parslow J., Takahashi M. (1997). Parameters of Photosynthesis: Definitions, Theory and Interpretation of Results. J. Plankton Res..

[54] Tilstone G., Figueiras F., Lorenzo L., Arbones B. (2003). Phytoplankton Composition, Photosynthesis and Primary Production during Different Hydrographic Conditions at the Northwest Iberian Upwelling System. Mar. Ecol. Prog. Ser..

[55] Relvas P., Barton E.D., Dubert J., Oliveira P.B., Peliz Á., da Silva J.C.B., Santos A.M.P. (2007). Physical Oceanography of the Western Iberia Ecosystem: Latest Views and Challenges. Prog. Oceanogr..

[56] Chassot E., Bonhommeau S., Dulvy N.K., Mélin F., Watson R., Gascuel D., Le Pape O. (2010). Global Marine Primary Production Constrains Fisheries Catches. Ecol. Lett..

[57] Andrade C. (1990). O Ambiente de Barreira Da Ria Formosa (Algarve, Portugal). Ph.D. Thesis.

[58] Cloern J.E. (1987). Turbidity as a Control on Phytoplankton Biomass and Productivity in Estuaries. Cont. Shelf Res..

[59] Morel A., Smith R.C. (1974). Relation between Total Quanta and Total Energy for Aquatic Photosynthesis. Limnol. Oceanogr..

[60] Kirk J.T.O. (2013). The Total Radiant Energy, and the Average Depth of All the Photons, in the Water Column. Am. Soc. Limnol. Oceanogr..

[61] Jacobs P., Kromkamp J.C., Van Leeuwen S.M., Philippart C.J.M. (2020). Planktonic Primary Production in the Western Dutch Wadden Sea. Mar. Ecol. Prog. Ser..

[62] Eilers P.H.C., Peeters J.C.H. (1988). A Model for the Relationship between Light and Intensity and the Rate of Photosynthesis in Phytoplankton. Ecol. Modell..

[63] Parsons T.R., Maita Y., Lalli C.M. (1984). A Manual of Chemical and Biological Methods for Seawater Analysis.

[64] Hansen H.P., Koroleff F., Grasshoff K., Kremling K., Ehrhardt M. (1999). Determination of Nutrients. Methods of Seawater Analysis.

[65] Borcard D., Gillet F., Legendre P. (2018). Canonical Ordination.

[66] Oksanen J., Simpson G.L., Blanchet F.G., Kindt R., Legendre P., Minchin P.R., O’Hara R.B., Solymos P., Henry M., Stevens H. (2022). Vegan: Community Ecology Package. R Package Version 2.6-2. https://cran.r-project.org/Package=vegan.

[67] R Core Team R (2022). A Language and Environment for Statistical Computing. R Foundation for Statistical Computing, Vienna, Austria. https://www.r-project.org/.

[68] RStudio Team RStudio (2022). Integrated Development Environment for R. RStudio, PBC, Boston, MA, USA. http://www.rstudio.com/.

