# Supplementary material for: Seasonal and Spatial Variability of Phytoplankton Primary Production in a Shallow Temperate Coastal Lagoon (Ria Formosa, Portugal)
Source: Plants (Basel). 2022 Dec 14;11(24):3511. doi: 10.3390/plants11243511 (PMC9781219; doi:10.3390/plants11243511)
Supplement: Supplementary file 1 [file plants-11-03511-s001.zip › plants-2049846-supplementary.pdf]

Table S1 – Descriptive statistics (mean, median, minimum, maximum, standard deviation) for several variables in each sampling location. T – temperature (ºC), Im – mean light intensity in the mixed layer (μmol photons m^{-2} s^{-1}), Zeu – depth of euphotic zone (m), DIN – concentration of dissolved inorganic nitrogen (μM), DRP - concentration of dissolved reactive phosphorus (μM), DSi - concentration of dissolved silicon (μM), Chl-a – chlorophyll-a concentration (mg m^{-3}), PP – water column primary production (mg C m^{-3} d^{-1}), P^B_{max} – maximal biomass-specific primary production rate (mgC mgChla^{-1} h^{-1}), I_{opt} – optimal light intensity (μmol photons m^{-2} s^{-1}), α – photosynthetic efficiency (mg C (mg Chl$a$)$^{-1}$ h^{-1} (μmol photons m^{-2} s^{-1})$^{-1}$).

		mean	median	min	max	sd
**Inlet**	T	17.36	17.80	11.50	21.31	3.35
	I_m	261.65	250.74	30.04	563.60	182.24
	Z_{eu}	13.26	13.56	8.14	20.34	3.82
	DIN	2.52	2.02	0.39	5.52	2.05
	DRP	0.11	0.08	0.00	0.40	0.15
	DSi	1.67	1.00	0.00	4.00	1.61
	Chl-a	0.78	0.74	0.00	2.08	0.73
	PP	53.89	54.00	3.51	118.43	46.89
	P^B_{max}	5.43	4.89	1.49	10.2	3.38
	E_{opt}	252.56	249.16	186.48	313.11	45.11
	α	0.0249	0.0234	0.00	.07	0.29
**Navigational channel**	T	16.72	18.25	9.28	21.52	4.46
	I_m	207.52	156.18	12.81	624.30	190.93
	Z_{eu}	7.29	7.46	4.07	10.85	2.28
	DIN	3.245	2.26	0.45	7.18	2.76
	DRP	0.13	0.15	0.00	0.30	0.12
	DSi	3.07	2.45	0.00	7.20	3.08
	Chl-a	0.73	0.58	0.00	1.85	0.79
	PP	113.38	86.55	14.01	308.39	116.52
	P^B_{max}	24.40	6.56	3.06	113.78	43.89
	E_{opt}	325.12	345.31	227.50	389.21	62.06
	α	0.1464	0.0338	0.01	0.73	0.29
**Urban centre**	T	17.35	19.46	8.18	21.97	4.88
	I_m	281.87	304.47	24.53	531.10	170.51
	Z_{eu}	4.14	4.07	2.03	7.46	1.90
	DIN	9.33	8.78	3.39	15.85	4.31
	DRP	0.20	0.20	0.00	0.50	0.16
	DSi	4.39	5.50	0.00	8.90	3.33
	Chl-a	1.00	0.55	0.00	3.60	1.27
	PP	175.03	147.02	11.92	646.93	218.78
	P^B_{max}	15.24	10.26	0.50	51.27	17.01
	E_{opt}	324.68	302.52	260.74	527.07	60.45
	α	0.0567	0.0382	0.00	0.19	0.06
**Inner lagoon**	T	16.54	18.86	8.01	20.10	4.81
	I_m	294.85	313.38	22.63	708.60	213.59
	Z_{eu}	5.93	5.42	2.71	9.49	2.17
	DIN	3.82	3.53	0.73	7.36	2.79
	DRP	0.18	0.05	0.00	0.70	0.28
	DSi	4.53	3.70	0.00	11.40	4.65
	Chl-a	0.87	0.85	0.00	1.85	0.89
	PP	235.11	119.44	21.07	718.74	277.17
	P^B_{max}	13.43	4.56	0.56	47.59	18.18
	E_{opt}	303.85	293.32	200.79	463.84	96.25
	α	0.0628	0.0121	0.00	0.21	0.09

Table S2 - Descriptive statistics (mean, median, minimum, maximum, standard deviation) for several variables in each season. T – temperature (ºC), Im – mean light intensity in the mixed layer (µmol photons m^{-2} s^{-1}), Zeu – depth of euphotic zone (m), DIN – concentration of dissolved inorganic nitrogen (µM), DRP - concentration of dissolved reactive phosphorus (µM), DSi - concentration of dissolved silicon (µM), Chl-a – chlorophyll-a concentration (mg m^{-3}), PP – water column primary production (mg C m^{-3} d^{-1}), P^B_{max} – maximal biomass-specific primary production rate (mgC mgChla^{-1} h^{-1}), I_{opt} – optimal light intensity (µmol photons m^{-2} s^{-1}), α – photosynthetic efficiency (mg C (mg Chl*a*)$^{-1}$ h^{-1} (µmol photons m^{-2} s^{-1})$^{-1}$).

		**mean**	**median**	**min**	**max**	**sd**
**Spring**	T	19.27	19.45	17.80	20.40	1.20
	I_m	221.01	206.07	71.84	363.30	109.39
	Z_{eu}	7.3	6.8	2.7	16.3	4.64
	DIN	8.69	6.67	5.52	15.85	4.83
	DRP	0.25	0.25	0.20	0.30	0.05
	DSi	7.88	8.05	4.00	11.40	3.11
	Chl-a	2.01	1.85	0.74	3.60	1.18
	PP	210.12	169.25	104.20	397.76	128.80
	P^B_{max}	10.28	9.08	4.89	18.08	6.24
	E_{opt}	301.77	290.74	273.07	352.51	35.97
	α	0.0377	0.0383	0.02	0.06	0.00
**Summer**	T	19.21	19.54	17.69	20.09	1.06
	I_m	421.03	519.60	68.90	708.60	243.62
	Z_{eu}	5.8	5.4	2.0	13.6	3.65
	DIN	3.73	2.88	0.39	8.78	4.08
	DRP	0.48	0.45	0.30	0.70	0.17
	DSi	0.08	0.00	0.00	0.30	0.15
	Chl-a	1.02	1.11	0.18	1.66	0.76
	PP	448.12	477.66	118.43	718.74	283.42
	P^B_{max}	40.53	19.07	10.20	113.78	49.03
	E_{opt}	262.31	249.04	200.79	350.36	63.67
	α	0.2570	0.1122	0.07	0.73	0.32
**Autumn**	T	19.60	19.56	17.70	21.97	1.66
	I_m	212.09	263.15	12.81	504.30	176.58
	Z_{eu}	7.7	6.1	2.7	14.9	4.43
	DIN	3.77	3.00	0.64	9.23	3.16
	DRP	0.10	0.10	0.00	0.20	0.08
	DSi	2.38	1.95	0.00	6.40	2.54
	Chl-a	0.46	0.18	0.00	1.39	0.55
	PP	85.63	63.85	11.92	193.10	70.82
	P^B_{max}	14.19	6.56	0.50	51.27	18.89
	E_{opt}	316.70	310.03	215.45	427.07	62.55
	α	.0576	.0318	0.00	0.21	0.08
**Winter**	T	11.57	12.45	8.01	14.80	2.73
	I_m	206.68	188.96	100.42	359.37	95.41
	Z_{eu}	10.1	8.8	6.8	20.3	4.46
	DIN	4.75	2.78	1.61	14.07	4.30
	DRP	0.02	0.00	0.00	0.11	0.04
	DSi	4.05	3.10	0.80	8.40	2.63
	Chl-a	0.68	0.42	0.00	2.08	0.78
	PP	25.52	26.21	3.51	45.79	15.54
	P^B_{max}	3.34	3.28	1.49	5.33	1.11
	E_{opt}	298.91	273.55	186.48	277.36	95.52
	α	.0089	.0085	0.0000	.02	0.00
